# Reverse phase protein array identification of triple-negative breast cancer subtypes and comparison with mRNA molecular subtypes

**DOI:** 10.18632/oncotarget.19719

**Published:** 2017-07-31

**Authors:** Hiroko Masuda, Yuan Qi, Shuying Liu, Naoki Hayashi, Takahiro Kogawa, Gabriel N. Hortobagyi, Debu Tripathy, Naoto T. Ueno

**Affiliations:** ^1^ Department of Breast Medical Oncology, The University of Texas MD Anderson Cancer Center, Houston, TX, USA; ^2^ Morgan Welch Inflammatory Breast Cancer Research Program and Clinic, The University of Texas MD Anderson Cancer Center, Houston, TX, USA; ^3^ Department of Bioinformatics and Computational Biology, The University of Texas MD Anderson Cancer Center, Houston, TX, USA; ^4^ Department of Breast Surgical Oncology, Showa University Hospital, Tokyo, Japan

**Keywords:** triple-negative breast cancer, molecular subtype, functional, proteomics, mRNA microarray

## Abstract

**Background:**

Reverse phase protein array (RPPA) analysis, allows investigation of potential targets at the functional protein level,. We identified TNBC subtypes at the protein level using RPPA and compared them with mRNA molecular subtypes (TNBCtype, TNBCtype-4, and PAM50) that is unique in its availability of both RPPA and mRNA analyses.

**Methods:**

We classified the samples from 80 TNBC patients using both k-means and hierarchical consensus clustering analysis and performed Ingenuity Pathway Analysis. We also investigated whether we could reproduce the mRNA molecular subtypes using the RPPA dataset.

**Results:**

Both clustering methods divided all samples into 2 clusters that were biologically the same. The top canonical pathways included inflammation, hormonal receptors, and MAPK signaling pathways for the first cluster [“inflammation and hormonal-related (I/H) subtype”] and the GADD45, DNA damage, and p53 signaling pathways for the second cluster [“DNA damage (DD)-related subtype”]. Further k-means cluster analysis identified 5 TNBC clusters. Comparison between sample classification using the 5 RPPA-based k-means cluster subtypes and 6 gene-expression-based TNBCtype molecular subtypes showed significant association between the 2 classifications (p = 0.017).

**Conclusions:**

The I/H and DD subtypes identified by RPPA advance our understanding of TNBC’s heterogeneity from the functional proteomic perspective.

## INTRODUCTION

Triple-negative breast cancer (TNBC) is not a simple, homogeneous breast cancer subtype; it collectively describes cancers that do not express the well-known target receptors estrogen receptor (ER), progesterone receptor (PR), and HER2. Varied research has evaluated TNBC’s heterogeneity, establishing a consensus that TNBC is not a single entity but rather a biologically heterogeneous group [1–6]. In order to divide the heterogeneous TNBC into homogeneous subtypes, several groups have used gene expression analysis, an approach that allows comprehensive investigation of more than 20,000 genes and their expression patterns. Several TNBC subtype classifications have been derived by clustering analysis using gene expression patterns, such as PAM50 intrinsic subtypes [2, 6, 7], claudin-low subtype [8], Burstein et al’s 4 subtypes [9], and Lehmann and colleagues’ molecular subtypes [4, 10]. The weakest point of gene expression analysis is that we cannot evaluate the genes’ active status. Additional barriers to daily clinical use of gene expression analysis are its difficult methods of analysis and poor reproducibility.

In contrast, reverse phase protein array (RPPA) analysis could be easily adapted to the clinical setting for evaluating subtypes because it allows investigation of potential targets at the protein level and could lead to the development of immunohistochemical assays [11–13]. A further strength of RPPA is that it reflects protein function; proteins are the most actionable and druggable cellular components. Thus, we sought to determine whether functional proteomics can define molecular subtypes and accelerate personalized medicine. In this study, we used RPPA data for 80 TNBC patients to identify subtypes at the protein level, assessed the biological features of each subtype using Ingenuity Pathway Analysis (IPA), and compared duration of patient survival by subtype. For the 57 samples for which we have RPPA and mRNA analyses (a unique dataset), we also investigated whether there is correlation between the RPPA subtypes and the subtypes derived from messenger RNA (mRNA) analysis. Comparing these 2 modalities provides complementary information that compensates for the drawbacks of each: gene expression profiling does not allow evaluation of the genes’ function and activation status, while RPPA technology does not show the extent of proteome coverage, as there are generally fewer than 200 analytes. We here have created a valuable dataset in that both mRNA and RPPA analyses are available for the same 57 patient samples. According to our search, The Cancer Genome Atlas (TCGA) is the only other public dataset that has the same information.

## RESULTS

### Identification of 2 stable RPPA TNBC subtypes

**Figure 1:**
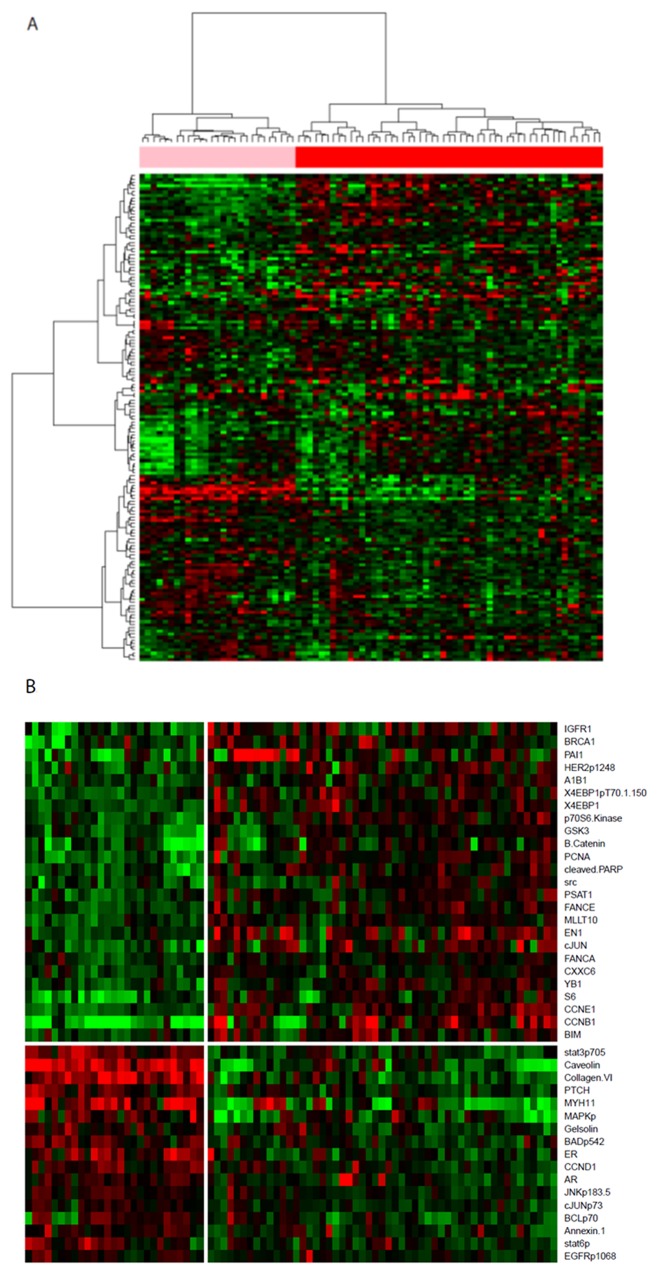
**(A)** Heatmap of unsupervised hierarchical clustering of TNBC samples with all protein expression data. Unsupervised k-means clustering separated samples into the same 2 clusters. **(B)** Heatmap showing the relative expression level of the 42 significantly differentially expressed proteins between the 2 stable subtypes.

The University of Texas MD Anderson Cancer Center previously created an RPPA database using samples from MD Anderson’s frozen breast tissue tumor bank; 80 patients from that dataset had triple-negative primary invasive ductal or invasive lobular breast carcinoma (n=3). For the current analysis, we first performed 2 types of clustering analysis, k-means and hierarchical, to classify the TNBCs into different molecular subtypes using the RPPA data. From both clustering analyses, we identified 2 stable clusters (Figure 1A) that were significantly different from each other (SigClust p = 0.012). Clusters of k = 2 from both clustering methods showed exactly the same subtypes (Supplementary Table 1), indicating that these 2 clusters were stable and sufficiently different.

**Figure 2:**
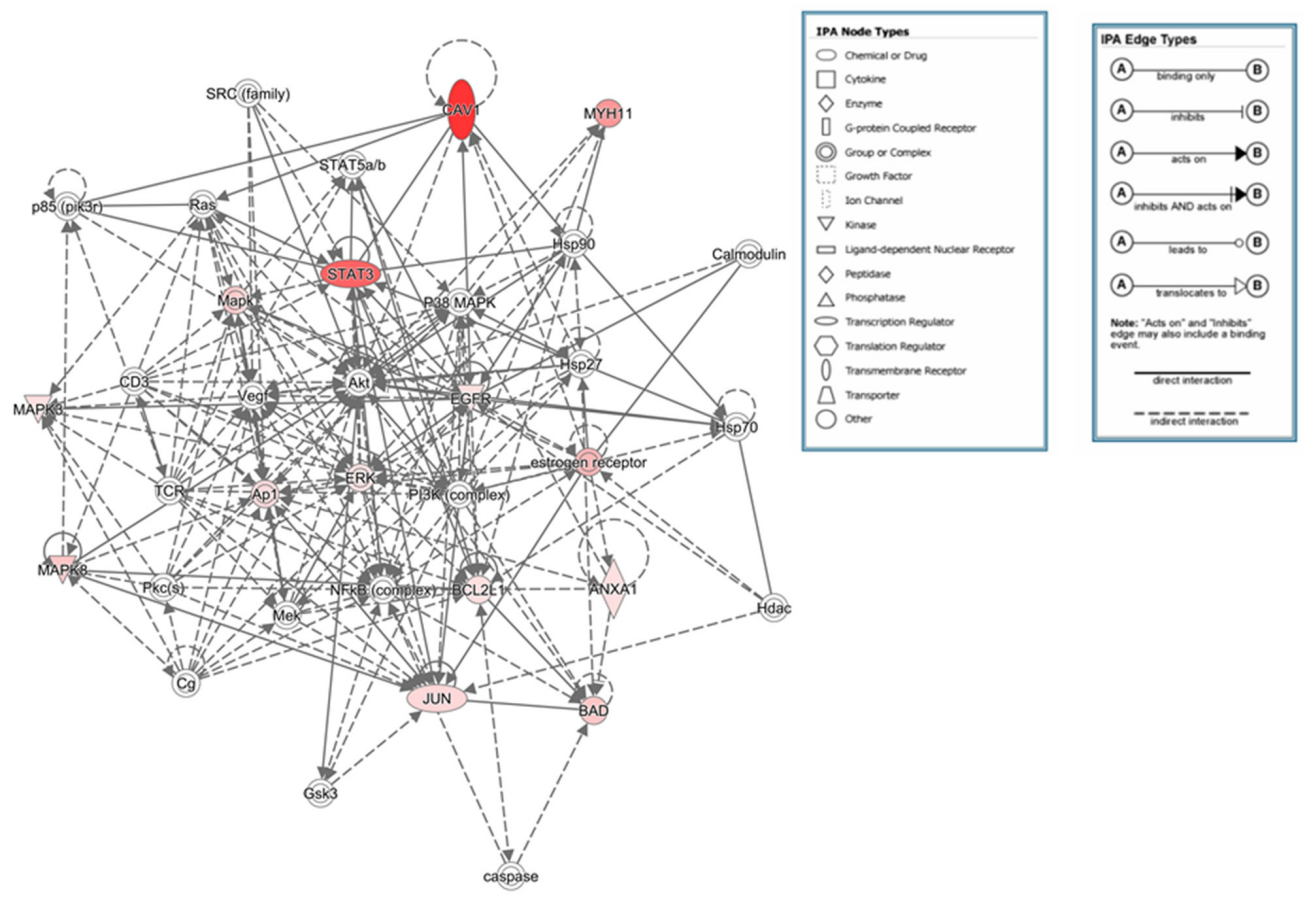
Top networks for the I/H-related subtype The genes in our list are labelled in red, indicating upregulation. The depth of color reflects the degree of upregulation; the deeper the color, the more upregulated the gene is.

**Figure 3:**
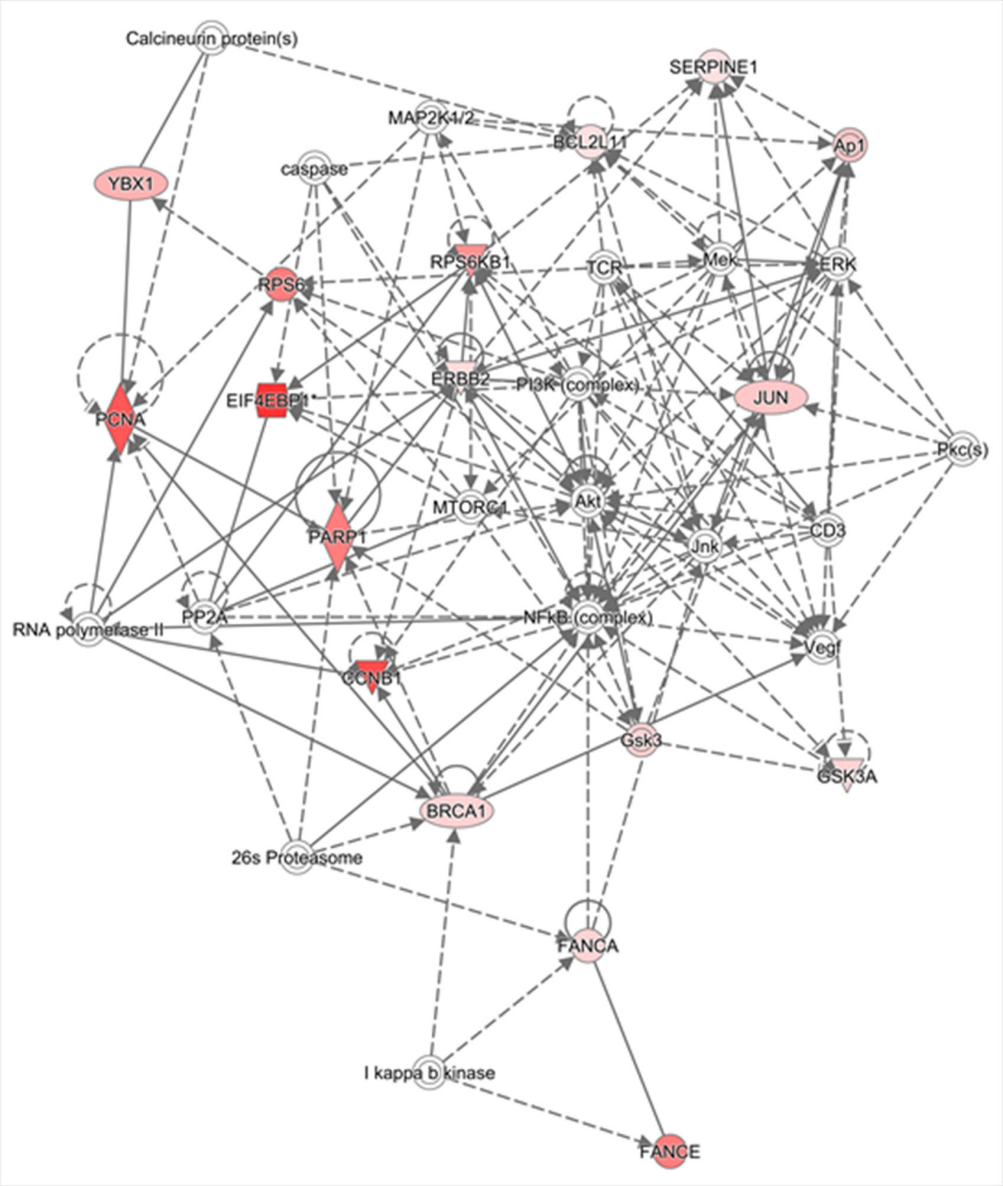
Top networks for the DD-related subtype See Figure 2 for description.

Protein signatures that were significant differentially expressed between the clusters were identified (Figure 1A, 1B and Table 1 ). Ingenuity pathway analysis of the protein signatures showed that the top canonical pathways associated with one of the clusters included the inflammation, hormonal receptor, and MAPK signaling pathways; we designated this cluster as the “I/H-related subtype” The canonical pathways associated with the other cluster included the Growth Arrest and DNA Damage (GADD45), DNA damage, and p53 signaling pathways; we designated this cluster as the “DD-related subtype” (Tables 1 and 2, Figures 2 and 3). The significant pathways were detected by IPA with highly significant p-values (10^-6^ - 10^-12^).

**Table 1:** Antibodies that were significantly differentially expressed between the 2 clusters identified by k-means and hierarchical clustering analysis of the RPPA results

Antibody name	Protein name	Gene symbol	Adjusted Bonferroni p-value
Higher expression in inflammation and hormonal subtype			
Caveolin 1	Caveolin 1	CAV1	3.05E-10
Collagen VI	Collagen VI	COL6A1	1.29E-09
stat3pS705	stat3 phosphorylation at S705	STAT3	3.02E-08
MYH11	MYH11	MYH11	3.28E-06
PTCH	Patche	PTCH1	6.58E-06
ER	Estrogen receptor alpha	ESR1	4.89E-05
JNKpT183/Y185	JNK phosphorylation at T183/Y185	MAPK8	0.000201828
BADp542	BAD phosphorylation at 542	BAD	0.000212416
c-JUNpS73	cjun N terminal kinase phosphorylation at S73	JUN	0.000986419
AR	Androgen receptor	AR	0.001376863
CCND1	Cyclin D1	CCND1	0.002897264
stat6pY641	Stat6 phosphorylation at Y641	STAT6	0.006211527
MAPKp	MAPK phosphorylation	MAPK3	0.007085294
Annexin.1	Annexin A1	ANXA1	0.009194289
BCLpS70	bcl2 phosphorylation at S70	BCL2L1	0.026207531
Gelsolin	Gelsolin	GSN	0.028422688
EGFRpY1068	EGFR phosphorylation at Y1068	EGFR	0.029595161
**Higher expression in DNA damage-related subtype**			
4EBP1	4E blinding protein1	EIF4EBP1	4.49E-08
CCNE1	Cyclin E1	CCNE1	9.18E-08
CCNB1	Cyclin B1	CCNB1	2.55E-07
PCNA	Proliferating cell nuclear antigen	PCNA	5.72E-07
S6	S6 ribosomal protein	RPS6	7.82E-06
Cleaved PARP	Cleaved PARP	PARP1	8.28E-06
FANCE	Fanconi anemia, complementation group E	FANCE	8.28E-06
P70 S6 Kinase	p70 S6 Kinase	RPS6KB1	3.17E-05
MILLT10	MILLT10	MLLT10	4.16E-05
4EBP1pT70	4EBP1 phosphorylation at T70	EIF4EBP1	0.000141
YB1	Y-box binding protein 1	YBX1	0.000274
cJUN	cJUN	JUN	0.001139
AlB1	Amplified in breast cancer 1	A1B1	0.001443
PSAT1	PSAT1	PSAT1	0.002198
BRCA1	BRCA1	BRCA1	0.002525
GSK3	Glycogen synthase kinase 3 beta	GSK3A	0.002525
IGFR1	Insulin-like growth factor receptor 1	IGFR1	0.002525
FANCA	Fanconi anemia, complementation group A	FANCA	0.002525
EN1	Engrailed-1	EN1	0.002644
HER2pY1248	HER2 phosphorylation at Y1248	ERBB2	0.004761
βCatenin	Beta catenin	CTNNB1	0.007731
PAI1	Plasminogen activator inhibitor-1	SERPINE1	0.013501
BIM	BIM	BCL2L11	0.013501
CXXC6	CXXC6	TET1	0.021359
Src	Src	SRC	0.047705

Shown are antibodies that were expressed at higher levels in the inflammation and hormonal subtype compared with the DNA damage-related subtype (top) and vice versa (bottom). A nonparametric Kruskal-Wallis test was used to identify proteins that were significantly differentially expressed. Proteins with an adjusted p-value by the Bonferroni method of less than 0.05 were considered significant.

**Table 2:** Top canonical pathways identified by Ingenuity Pathway Analysis (IPA) for each cluster

Cluster	Top canonical pathways
Inflammation and hormonal subtype	Pancreatic Adenocarcinoma Signaling Colorectal Cancer Metastasis Signaling Glucocorticoid Receptor Signaling UVB-Induced MAPK Signaling ErbB2-ErbB3 Signaling
DNA damage-related subtype	GADD45 Signaling Molecular Mechanisms of Cancer DNA Damage-Induced 14-3-3 Signaling Neuregulin Signaling p53 Signaling

Shown are the canonical pathways significantly associated with each cluster when the samples were divided into 2 clusters by k-means and hierarchical clustering. The significant pathways were detected by IPA with highly significant p-values **(**10^**-6**^ **-** 10^**-12**^).

We next compared whether these subtypes predict disease-free survival (DFS) and overall survival (OS). Kaplan-Meier analysis showed that patients’ OS and DFS rates, The I/H-related subtype showed better survival, but the differences between the two clusters were not significant. (DFS: p = 0.3; OS: p = 0.1) (Supplementary Figure 1).

### Further identification of RPPA TNBC subtypes and comparison with mRNA-derived subtypes

In order to determine whether there is a positive correlation between mRNA and proteomic classifications, we compared the RPPA subtypes and Lehmann et al’s original 6 mRNA molecular subtypes [4]. The latter 6 subtypes--basal-like 1 (BL1), basal-like 2 (BL2), immunomodulatory (IM), mesenchymal (M), mesenchymal stem-like (MSL), and luminal androgen receptor (LAR)--and an additional unstable (UNS) group were identified in 2011 on the basis of gene ontologies and differential gene expression [4], and we have confirmed these subtypes using the same methodology [14]. We also showed the correlation between these 6 subtypes and pathologic complete response [14]. In 2016, Lehmann et al published a preliminary analysis refining the subtypes to 4 [10]. However, further definition of these subtypes is needed before they can be exploited for targeted therapy or prognostic prediction in a clinical setting. Thus, we focused on k-means clustering analysis, which was originally used to identify the 6 subtypes. Consensus clustering with the k-means clustering method for each round was carried out to identify clusters in the RPPA dataset. Core samples for each cluster were selected based on positive silhouette width for each cluster, indicating the samples were closer to samples of the same cluster than to samples in other clusters. The total number of samples was reduced to 74 core samples.

**Figure 4:**
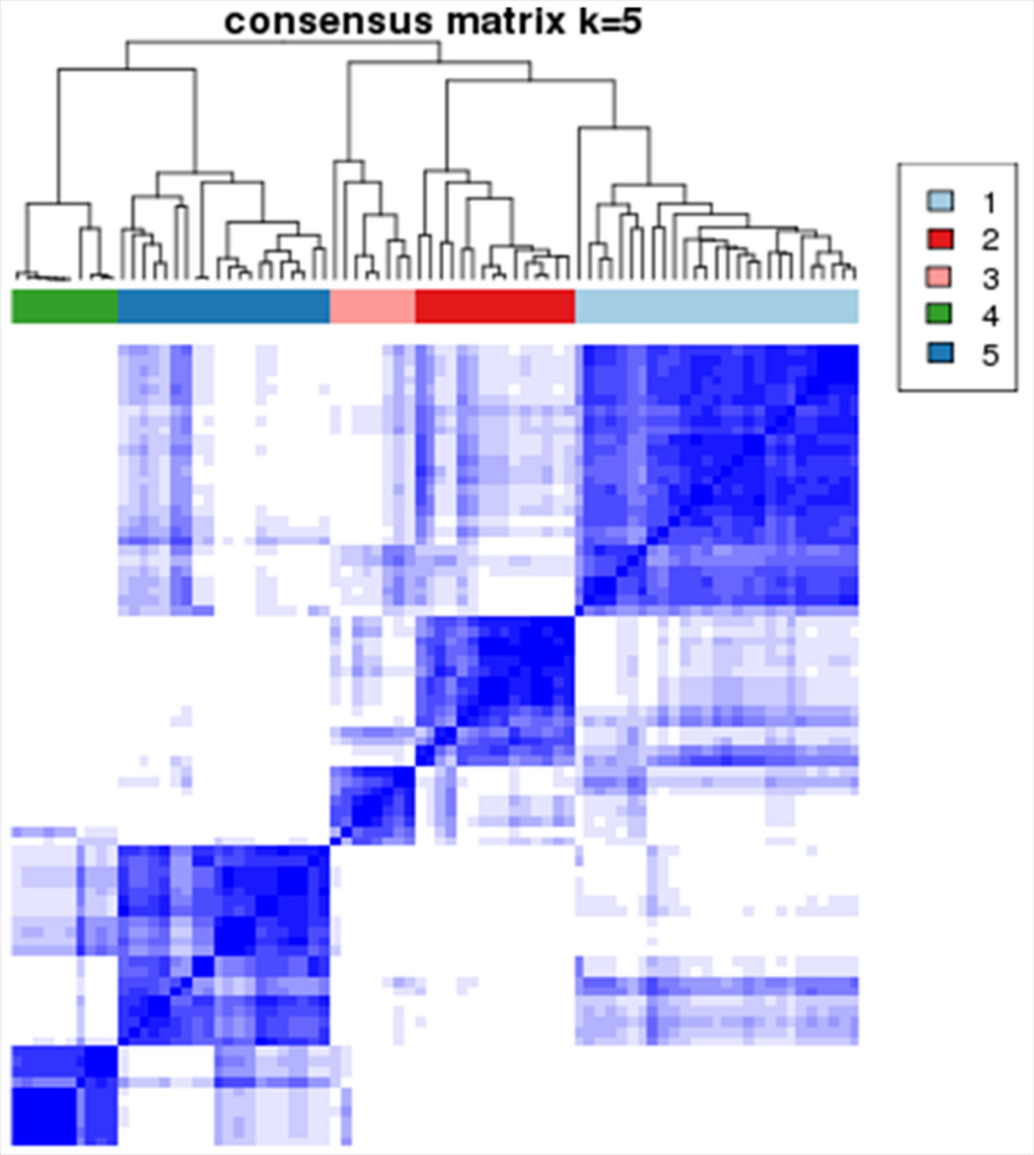
Consensus clustering analysis results displaying the robustness of sample classification using multiple iterations of k-means clustering In this heatmap of the clustered consensus matrix, rows and columns of the matrix both indicate samples. The darkness of color in the heatmap represents how frequent 2 samples are clustered together. The darker, the more frequent. This heatmap clearly shows a pattern of 5 compact groups of samples frequently clustered together along the diagonal. The color bar on top indicates 5 clusters of samples.

**Figure 5:**
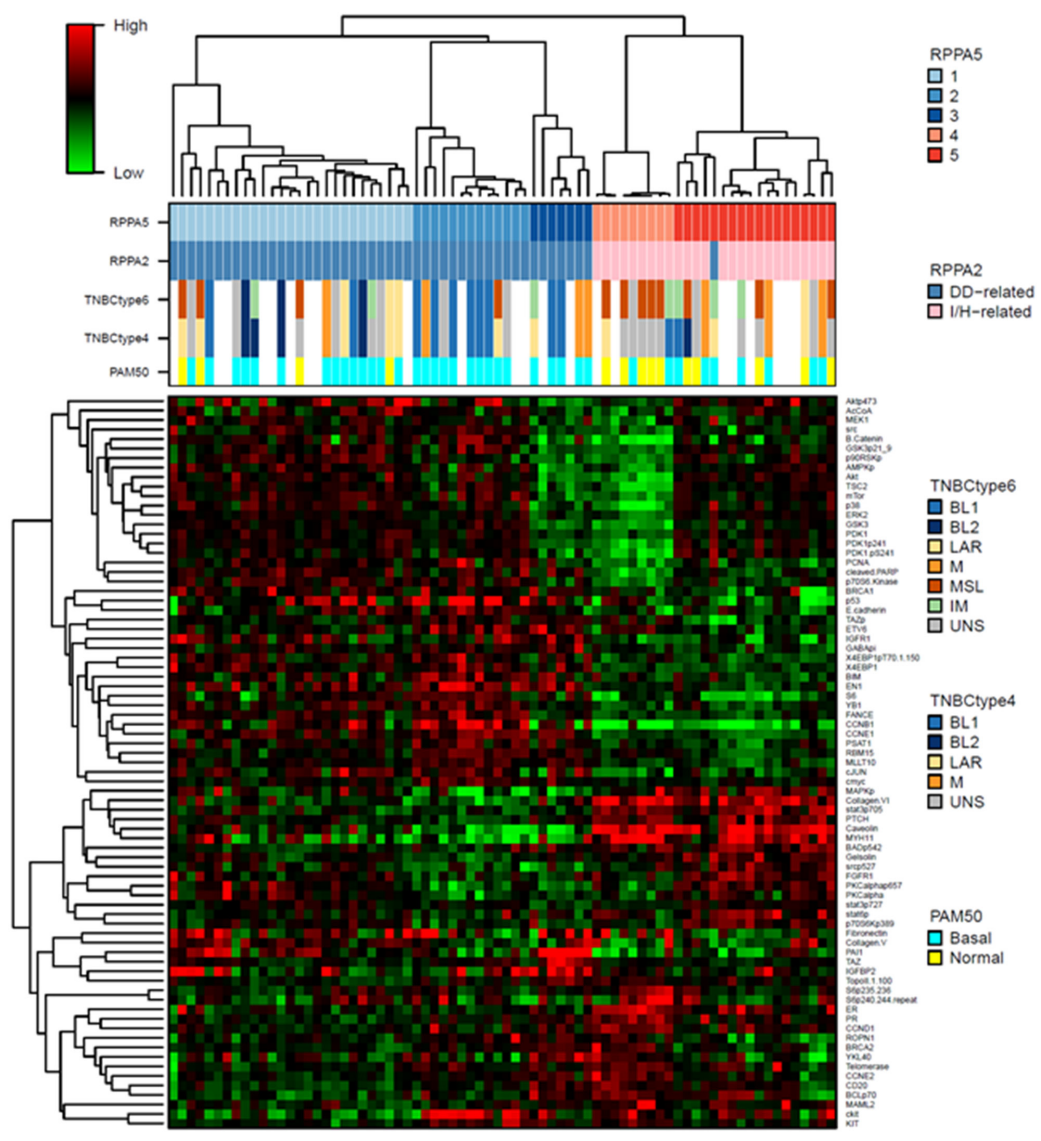
Heatmap of the RPPA 5-cluster-based signature proteins The dendrogram on top shows the consensus clustering of the 74 core RPPA samples. Classifications of these samples using different methods are shown below the dendrogram. The proteins shown are the 77 PAM signature proteins for the 5 RPPA clusters, with median centered expression values.

Consensus k-means clustering analysis of the core samples allowed us to further identify 5 clusters (Table 3) in the RPPA data statistically (Figure 4). The heatmap for the clustered consensus matrix (Figure 5A) clearly shows 5 compact groups of samples frequently clustered together along the diagonal. Five was selected as the optimal cluster number as it is at the point in which the relative change in area under the cumulative distribution function (CDF) plot did not differ from random changes with increasing cluster number (Supplementary Figures 2 and 3).

**Table 3:** Patient characteristics for the full cohort (74 core samples) and the 5 clusters identified by k-means analysis of the RPPA dataset

		Entire cohort	Cluster 1	Cluster 2	Cluster 3	Cluster 4	Cluster 5	P-value
Total no. of patients		74	27	13	7	9	18	
Age	≤50 years	37	12	7	3	5	10	0.937
	>50 years	37	15	6	4	4	8	
Menopausal status	Premenopausal	44	16	7	5	6	10	0.941
	Postmenopausal	30	11	6	2	3	8	
Tumor T classification	T0	1	0	0	1	0	0	0.03
	T1	18	5	2	1	3	7	
	T2	37	18	5	1	5	8	
	T3	9	2	1	3	0	3	
	T4	8	2	4	1	1	0	
	Unknown	1	0	1	0	0	0	
Lymph node metastasis status	Negative	40	14	8	3	5	10	0.943
	Positive	33	13	5	4	4	7	
	Unknown	1	0	0	0	0	1	
Nuclear grade	1	1	0	0	0	0	1	0.428
	2	6	2	0	1	0	3	
	3	67	25	13	6	9	14	
Neoadjuvant chemotherapy	No	45	16	7	3	7	12	0.65
	Yes	29	11	6	4	2	6	
Adjuvant chemotherapy	No	30	13	4	4	3	6	0.667
	Yes	44	14	9	3	6	12	
Neoadjuvant radiotherapy	No	71	26	13	5	9	18	0.047
	Yes	3	1	0	2	0	0	
Adjuvant radiotherapy	No	26	8	5	3	6	4	0.223
	Yes	48	19	8	4	3	14	

P-values are the result of ANOVA test (for age as a continuous variable) or Fisher’s exact test (for all other categorical clinical variables) comparing each clinical variable between the 5 clusters.

These 5 clusters are further divisions of the previous 2 clusters we identified (DD-related subtype, clusters 1, 2, 3; I/H-related subtype, clusters 4, 5) (Figure 5), with the exception of only 1 sample.

Comparison between sample classification using the 5 RPPA-based k-means clusters (1-5) and Lehmann et al’s 6 gene-expression-based molecular subtypes showed significant association (Fisher’s exact test, p = 0.017) (Table 4A). Subtype BL2 was RPPA cluster 1-like (p = 0.037), meaning most BL2 tumors were in cluster 1, although cluster 1 contained other subtypes. Cluster 2 was BL1-like (p = 0.002), and cluster 4 was MSL-like (p = 0.005). All BL1 and BL2 patients belonged to the DD-related subtype, indicating that the basal subtype showed high reproducibility regardless of the modality we used to identify these subtypes.

**Table 4A:** Comparison of sample classification according to 5 RPPA clusters and 6 gene-expression-based TNBC subtypes for the 52 patients with available data

RPPA cluster	BL1	BL2	IM	LAR	M	MSL	UNS	p-value
1	2	3	2	3	1	3	4	0.017
2	6	0	0	0	1	1	2	
3	2	0	1	0	2	0	0	
4	0	0	1	0	0	5	1	
5	0	0	3	1	3	3	2	

P-value shown is the result of Fisher’s exact test for association between the 2 classification results.

Significantly enriched:

BL2 was enriched in RPPA-cluster-1 (p = 0.037)

RPPA cluster 2 was enriched in BL1 (p = 0.002).

RPPA cluster 4 was enriched in MSL (p = 0.005).

However, when we focused on each cluster, the most correlation was seen in only the BL1/2 subtypes, and we couldn’t identify the 6 molecular subtypes using functional proteomics analysis.

Lehmann et al refined TNBC molecular subtypes from 6 (TNBCtype) into 4 (TNBCtype-4) tumor-specific subtypes (BL1, BL2, M, and LAR) because they found significant evidence that the IM and MSL TNBC subtypes represent tumors with substantial numbers of infiltrating lymphocytes and tumor-associated mesenchymal cells, respectively. [10]

In order to investigate the biological features of our 5 RPPA clusters more accurately and determine the positive correlation with mRNA molecular subtypes, we also compared our 5 RPPA clusters with TNBCtype-4. When the RPPA clusters were compared with TNBCtype-4, there was a marginal significant association (Fisher’s exact test, p = 0.07) (Table 4B). Focused on each subtype, RPPA cluster 2 was enriched in TNBCtype-4 subtype BL1 (p = 0.006), and TNBCtype-4 subtype BL2 was enriched in RPPA cluster 1 (p = 0.043). There was no significant association between the TNBCtype-4 LAR or TNBCtype-4 M subtypes and the RPPA clusters.

**Table 4B:** Comparison of sample classification according to 5 RPPA clusters and 4 gene-expression-based TNBC subtypes for the 52 patients with available data

RPPA cluster	BL1	BL2	LAR	M	UNS	p-value
1	2	4	5	1	6	0.07
2	6	0	1	1	2	
3	2	0	1	2	0	
4	1	0	1	0	5	
5	1	1	2	3	5	

P-value shown is the result of Fisher’s exact test for association between the 2 classification results.

Significantly enriched:

RPPA cluster 2 was enriched in TNBCtype4 BL1 (p = 0.006).

TNBCtype4 BL2 was enriched in RPPA cluster 1 (p = 0.043).

We also compared our 5 RPPA cluster with the PAM50 subtypes, reported by Perou et al. [2, 6, 15, 16], which is well known and accepted in the clinical setting. When compared with the PAM50 classification, RPPA cluster 2 was enriched in the basal subtype (p = 0.046), and RPPA cluster 4 was enriched in the normal-like subtype (p = 0.011) (Table 4C).

**Table 4C:** Comparison of sample classification according to 5 RPPA clusters and PAM50 subtypes for the 52 patients with available data

RPPA cluster	Basal	Normal-like
1	14	4
2	10	0
3	5	0
4	2	5
5	7	5

Significantly enriched:

RPPA cluster 2 was enriched in the basal subtype (p = 0.046).

RPPA cluster 4 was enriched in the normal-like subtype (p = 0.011)

The OS rates significantly differed between the 5 RPPA clusters (log-rank test, p = 0.002). However, this result was affected by patient characteristics; most patients with T4 disease belonged to clusters 2 and 1 (Supplementary Figure 2). The DFS plot did not show a significant difference between subtypes (Supplementary Figure 2). We listed the pattern of protein expression in each of the 5 RPPA clusters (Supplementary File 1).

## DISCUSSION

In this study, we identified 2 major stable clusters using functional proteomics, and pathway analysis identified the biological features of these 2 groups as DD-related and I/H-related subtypes. Two types of clustering analysis produced the same results; thus, these clusters can be considered promising and stable. When compared with the mRNA molecular subtypes, RPPA cluster 1 and mRNA subtype BL-2 (in both the TNBC 6 subtype and TNBCtype-4 classifications), and RPPA cluster 2 and BL1 (in both TNBC 6 subtype and TNBCtype-4), showed strong positive correlation, and most of these tumors belonged to the DD-related RPPA subtype and the basal-like PAM50 subtype, indicating that the basal and non-basal subtypes showed high reproducibility regardless of the modality used to identify them. However, we couldn’t discriminate the other molecular subtypes using functional proteomics analysis. The results may indicate that classification is discordant for different modalities such as functional proteomics analysis and mRNA molecular analysis or that some subtypes described by mRNA are not reliable.

The two identified subtypes suggest the potential for different treatment strategies. From the DD-related subtype’s biological features, platinum agents such as cisplatin and carboplatin could potentially have efficacy for this subtype due to overexpression of cell cycle and DNA damage response gene signatures. For the I/H-related subtype, androgen receptor (AR) showed high expression and IPA identified hormonal signaling pathways. As previously suggested by Burstein et al’s and Lehmann et al’s findings, even within TNBC there is a subtype that shows high expression of ER and AR [4, 9]. The Cancer Genome Atlas (TCGA) Network analyzed breast cancers using RPPA and defined a luminal tumor subtype that showed high ER, AR, and BCL2 protein expression [17]. Thus, the I/H-related subtype has a similar biology to that of the luminal subtype and AR inhibitor may have potential to treat this subtype. Further, MAPK or Stat3 signaling was another top canonical pathway for the I/H-related subtype; thus, anti-inflammatory drugs such as COX2 inhibitors, which inhibit MAPK or Stat3 signaling [18–20], have potential to be targeted drugs for this subtype. We believe that defining subtypes will enable development of subtype-specific targeted therapies and accelerate personalized medicine.

For the DD-related subtype, the top canonical pathways were GADD45 Signaling, Molecular Mechanisms of Cancer, and DD-Induced 14-3-3 signaling. In this subtype, basal-like feature genes, such as CCNE1, PARP1, and BRCA1, showed high expression.

Interestingly, STAT3, JNK, and cJUN were highly expressed in luminal and our I/H-related subtype, and these proteins have an important role in inflammation. Moreover, the MAPK signaling pathway was one of the top canonical pathways of the I/H-related subtype because of the high expression of JUN, BAD, MAPK3, MAPK8, BCL2L1 and EGFR proteins. Thus, the biological features of this subtype also include inflammation. We showed high protein expression of caveolin-1 and collagen VI in the I/H-related subtype, but as the TCGA group reported, it is possible that these proteins were not specific to tumor but were produced by the microenvironment and/or cancer-activated fibroblasts [17].

Functional proteomics classification also indicated 2 types of basal subtype; especially notable was that 4 of the 5 BL2 patients in the TNBCtype-4 classification belonged to RPPA cluster 1. Further, cluster 1 (similar to BL2) showed high levels of FGFR and PI3K-AKT signaling pathway-related proteins. Our previous study also showed differences in pathologic complete response rates for patients with the 2 basal subtypes (BL1 was associated with the highest pCR rate and BL2 the lowest) even though basal-like subtype in intrinsic subtype reported favorable chemosensitivity, might be related to the expression of growth factors and proliferation genes [14]. When we compared the RPPA clusters with the PAM50 classification, all RPPA cluster 2 (similar to BL1) tumors belonged to the basal-like subtype, but not all RPPA cluster 1 (similar to BL2) tumors did. Thus, functional proteomics could possibly be used to identify the chemotherapy-resistant subset of the basal-like type. It is truly requisite for TNBC patients that some patients showed progressive disease during neoadjuvant chemotherapy.

Only one other report has classified TNBC using proteomics. Lawrence et al. reported the proteomic landscape of TNBC in 2015 [21]; they performed quantitative proteomics analysis of 20 human-derived breast cell lines and 4 primary breast tumors to a depth of more than 12,000 distinct proteins. They identified 2 overarching groups containing 4 clusters using hierarchical clustering analysis. They determined genetic abnormalities in each cluster and compared them with the mRNA classification, then labeled these clusters as luminal, basal-like 2, basal-like 1, and mesenchymal-like/claudin-low subtypes. The results were similar to ours in that they also identified 2 basal-like subtypes. However, when they focused on the cancer-signaling proteins associated with each subtype, they found that despite overall concordance of whole proteome profiles with various cellular phenotypes, in most cases the expression of particular cancer proteins was not uniformly characteristic of one subtype or another.

Over the past 15 years, several mRNA expression-guided TNBC classifications have been reported. Some parts of these molecular classifications are biologically similar, have high reproducibility, and have shown clinical relevance, and thus we are beginning to understand true TNBC subtypes on the basis of their specific biology, but the various subtypes do not match perfectly across classifications. Further refinement is needed before these methodologies can be adapted into daily practice. Current barriers to use of mRNA-guided subtyping include 1) the complicated analysis, which requires a high level of skill; 2) the low reproducibility; and 3) the need to confirm true clinical relevance. Moreover, since among TNBCs we have not yet found target genes as clear and consistent as ER, PR, and HER2, currently we have to investigate a huge number of genes’ expression and mutations and identify subtypes according to their distributions and relationships. Because gene expression takes the form of continuous variables, clinical application relies heavily on the interpretation of data to establish thresholds, with the risk of subjectivity [17]. Thus, there is a risk of classifying TNBC subtypes based on chance rather than their true clinical and/or biological differences. Others have noted the need to classify cancers not only according to their molecular profiles but also based on their response to therapies. Given these current approaches, in order to adapt these findings to clinical practice, we still have many challenges.

Confirming the subtype findings using multiple modalities is one solution to determine highly credible subtypes. As we seek to adapt subtypes into clinical practice, proteomics has the potential to overcome the inability of gene analysis to investigate biological features directly. Through the comprehensive analysis of protein expression levels and activation statuses, proteomics can enable investigation of cancer pathogenesis and biology and can lead to the development of immunohistochemical assays. We tested 154 total and phosphorylated proteins in this study; of those we selected 42 proteins to identify 2 clusters and 61 proteins to identify 5 clusters, this is still a large number of proteins for clinical practice, but it has potential to be accepted in daily clinical practice compared with mRNA expression. The RPPA approach permits, with just a small amount of material, quantification of the expression level and modification of proteins as a continuous value for a large number of patients [11].

The identification of homogeneous TNBC subtypes is not as simple as classifying ER-positive breast cancer, due to the lack of powerful biomarkers that distinguish each subtype. We need not only to identify strong biomarkers but also a biology-oriented comprehensive approach to identify optimal TNBC subtypes that improve the success rate of new targeted therapies due to optimal patient selection and result in better prognosis. However, we still do not know the optimal modality for identifying the subtypes and how to interpret the discordance that arises in identifying subtypes using different methods [22]. Our study is an important first step to defining the differences between the results of proteomic and mRNA classification and clarifying the issues involved in establishing a standard classification method using functional proteomics.

However, there were several limitations of this study. First, the samples were collected retrospectively, and thus clinical situations may have led to bias in terms of factors such as treatment selection and the duration of sample storage. Although the number is not big enough to statistically affect our findings, there are 3 lobular cancer patients included in this study. Second, as noted above, the RPPA did not cover all of the proteins corresponding to the genes of the mRNA expression assays. Third, the merit of functional proteomics in investigating phosphorylation disappeared in the course of our pathway analysis because we converted both total and phosphorylated proteins to the same gene symbol. These limitations might have prevented us from identifying more specific TNBC subtypes. For example, our subtype encompassing inflammation- and hormonal-related biological features couldn’t distinguish these 2 features.

We believe that functional proteomics is one of the strategies that will help us to achieve the goal of overcome TNBC heterogeneity and explore personalized medicine. A strength of our study is that we had data for both mRNA and RPPA analysis in the same patients so that we were able to compare and validate the modalities’ concordance. Although we found some discordance between the mRNA and proteomic classifications, our study confirmed that both methods divide basal and non-basal subtypes and showed potential for distinguishing more detailed TNBC subtypes such as basal 1 and 2 and the inflammation and hormonal subtype. For future investigation, collection of multiple specimens for patients over time is needed, even for patients receiving standard care in community clinics, in order to accelerate personalized therapy development in TNBC based on biology-oriented comprehensive approaches. These efforts could improve the sensitivity, specificity, and predictive power of TNBC subtypes and yield optimal treatment for these heterogeneous breast cancers.

## MATERIALS AND METHODS

### Patient samples

As described in our group’s previous publication [23], the tumor samples for the RPPA database were obtained from biopsies from patients with primary invasive ductal or invasive lobular breast carcinoma who underwent surgery prior to any systemic therapy between June 1992 and March 2007 at MD Anderson Cancer Center. Patients with ductal carcinoma in situ, metaplastic carcinoma, or sarcoma were excluded. All specimens were collected and analyzed under institutional review board approval. The current analysis consisted of the 80 patients from that dataset who had TNBC.

### RPPA data

RPPA analysis was performed in our laboratory as described previously [13, 23–25]. Briefly, tumor lysates were normalized to 1 μg/μL concentration using a bicinchoninic acid assay. The lysates were then boiled with 1% sodium dodecyl sulfate, and the supernatants were manually diluted in 6 or 8 2-fold serial dilutions with lysis buffer. An Aushon Biosystems 2470 arrayer was used to create sample arrays from the serial dilutions on nitrocellulose-coated FAST slides (Schleicher & Schuell BioScience, Inc.). The slides were analyzed and protein expression quantitated with the use of Microvigene software (VigeneTech Inc., Carlisle, MA).

The RPPA dataset consists of expression levels for 108 full proteins as well as 46 phosphorylated (phospho) proteins, and these data were normalized using the load-control method. The RPPA data before and after load-control normalization are provided in Supplementary Files 2 and 3. The antibody names of the RPPA dataset (whether full proteins or phosphoproteins) were mapped to HGNC (HUGO Gene Nomenclature Committee) gene symbols and used in downstream pathway analysis. There were 112 unique gene symbols for the RPPA dataset. The different forms of antibodies (full proteins or multiple phosphorylated proteins) for each gene symbol were treated as multiple probes measuring for the same gene.

### Matching mRNA expression data

Fifty-two of the 74 RPPA TNBC core samples had matching mRNA expression profiles. Core samples for each cluster were selected based on positive silhouette width for each cluster, indicating the samples were closer to samples of the same cluster than to samples in other clusters.

The mRNA expression data were measured using Affymetrix microarray HG-U133A. The robust multi-array analysis (RMA) method was used to normalize the mRNA expression data, and BioConductor package hgu133a.db was used to annotate probe sets. Genes with multiple probe sets were collapsed by taking the mean.

### Clinical data

As described previously [23], clinical data were extracted from MD Anderson’s Breast Cancer Management System database. HER2 levels were evaluated as continuous variables by RPPA analysis rather than as positive or negative by immunohistochemistry and/or fluorescence in situ hybridization.

### Statistical analysis

Consensus clustering with k-means and hierarchical clustering methods was carried out using the R package ConsensusClusterPlus [26] to identify clusters in the RPPA dataset. Core samples for each cluster were selected based on positive silhouette width for each cluster. Signature genes of the clusters were identified using the PAM method [27], at a median false discovery rate of 0.01. For the initial 2 large clusters, the k-means and hierarchical clustering were carried out using R packages kmeans and ClassDiscovery: Mosaic (http://bioinformatics.mdanderson.org/main/OOMPA:Overview). A nonparametric Kruskal-Wallis test was used to identify proteins that were significantly differentially expressed. Proteins with an adjusted p-value of less than 0.05 using the Bonferroni method were considered significant. Significance testing between clusters were carried out using R package SigClust [28]. Kaplan-Meier plots were used for overall survival (OS) and disease-free survival (DFS) analysis. ANOVA and Fisher’s exact test were used to compare clinical variables between clusters. Association testing between the samples’ classifications by RPPA-based clusters and gene-expression-based subtypes was also carried out using Fisher’s exact test.

QIAGEN’s Ingenuity pathway analysis (IPA^®^, QIAGEN Redwood City, http://www.ingenuity.com/) was used to identify canonical pathways that were significantly associated with each cluster. The IPA program collapsed all protein forms (“total” and “phosphorylated”) into unique gene symbols by using the maximal value among the different forms.

The normalized and annotated gene expression data were fed to the online server TNBCtype (http://cbc.mc.vanderbilt.edu/tnbc/prediction.php) to predict molecular subtypes in these TNBC samples. The algorithm for predicting the updated TNBCtype-4 subtypes was obtained from Brian Lehmann (Vanderbilt-Ingram Cancer Center). Samples classified as IM or MSL were reassigned to subtype BL1, BL2, M, or LAR to which highest correlation was obtained using the 6-subtype output. Samples with p-value greater than 0.05 or a difference in correlation coefficients between the highest and 2^nd^-highest subtypes of less than 0.05 were assigned as “UNS” (unstable). We implemented this algorithm using R. The normalized gene expression data were also used to predict PAM50 subtype [16].

## SUPPLEMENTARY MATERIALS FIGURES AND TABLE








