# Supplementary material for: Reverse phase protein array identification of triple-negative breast cancer subtypes and comparison with mRNA molecular subtypes
Source: Oncotarget. 2017 Jul 31;8(41):70481–95. doi: 10.18632/oncotarget.19719 (PMC5642571; doi:10.18632/oncotarget.19719)
Supplement: Supplementary file 1 [file oncotarget-08-70481-s001.pdf]

Reverse phase protein array identification of triple-negative breast cancer subtypes and comparison with mRNA molecular subtypes

SUPPLEMENTARY MATERIALS

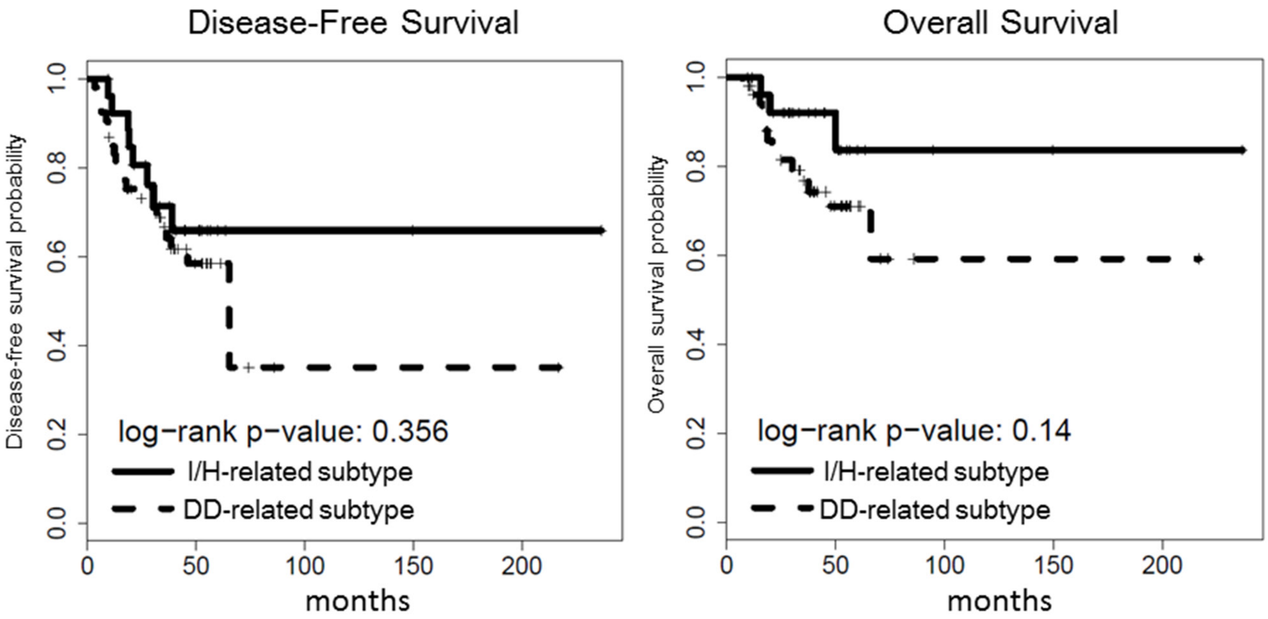

Supplementary Figure 1: Kaplan-Meier plots of disease-free survival (DFS; left) and overall survival (OS; right) for the 74 TNBC patients according to the 2 RPPA clusters.

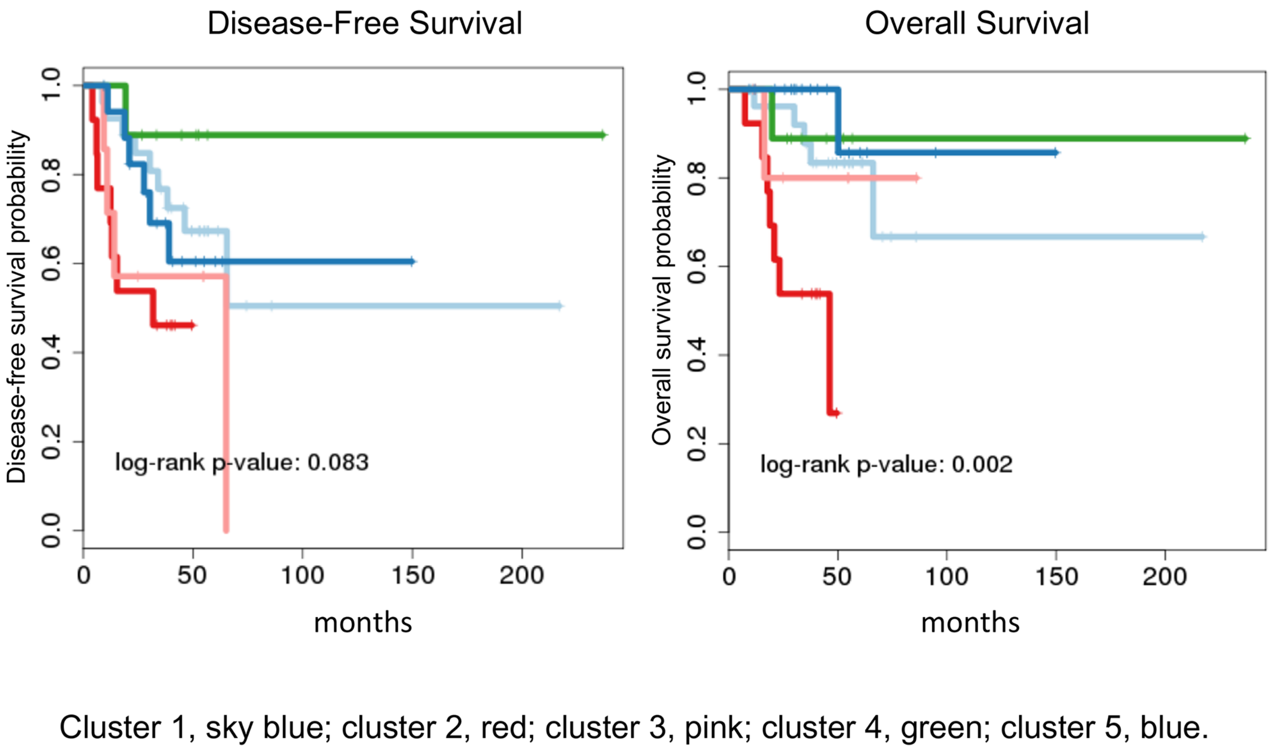

Supplementary Figure 2: Kaplan-Meier plots of disease-free survival (left) and overall survival (right) for the 74 TNBC patients divided into the 5 clusters defined by k-means clustering. Cluster 1, sky blue; cluster 2, red; cluster 3, pink; cluster 4, green; cluster 5, blue.

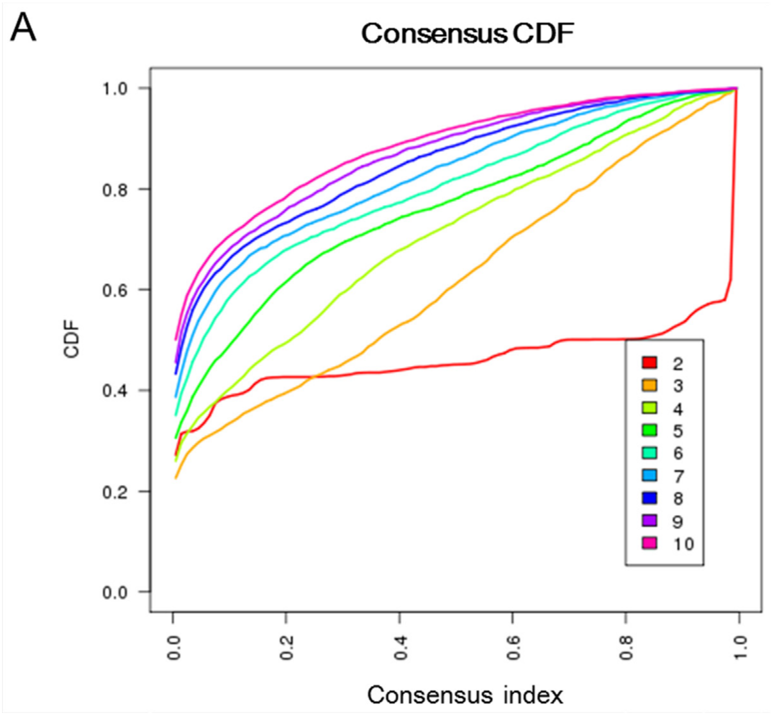

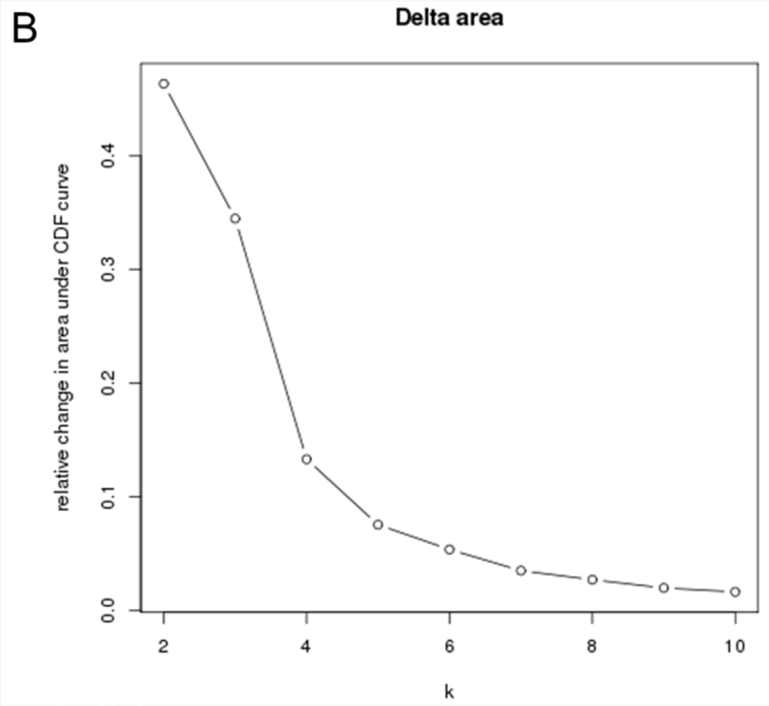

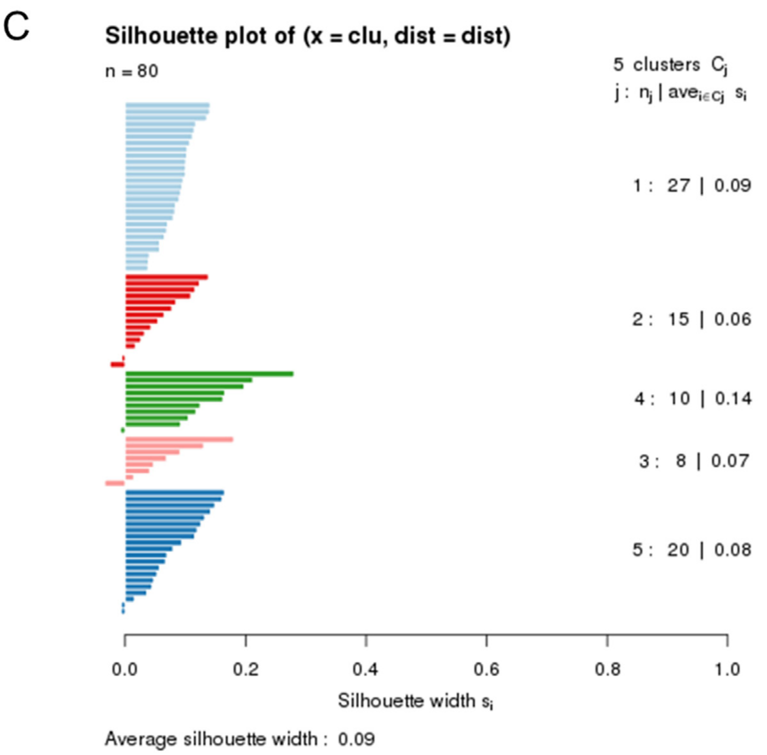

Supplementary Figure 3: Consensus clustering statistics. The **(A)** cumulative distribution function (CDF) of consensus and **(B)** the relative changes of area under the CDF curve over the number of clusters. The optimal cluster number is at the point at which the relative increase in area under the CDF curve does not differ from random changes with increasing cluster number. Thus, the optimal cluster number was 5. **(C)** Silhouette width plot showing the core samples (horizontal bars with positive silhouette widths) of each cluster.

Supplementary Table 1: Comparison of the two stable RPPA TNBC clusters identified using k-means and hierarchical clustering algorithms. Both methods showed the same separation of samples into two clusters, only the naming of the clusters switched

	Hierachical clusters	
**K-means clusters**	**1**	**2**
1	0	27
2	53	0

Supplementary File 1: RPPA 5 cluster pattern of protein expression

See Supplementary File 1

Supplementary File 2A: RPPA data before load-control normalization.

See Supplementary File 2

Supplementary File 2B: RPPA data after load-control normalization

See Supplementary File 3
